# Radiation Exposure and Cancer Mortality in Populations Living Around the Semipalatinsk Nuclear Test Site in Kazakhstan

**DOI:** 10.3390/ijerph23081037

**Published:** 2026-08-09

**Authors:** Altay Dyussupov, Rauan Kissina, Nailya Chaizhunussova, Dariya Shabdarbayeva, Andrey Orekhov, Zhanargul Smailova, Meruyert Massabayeva, Assel Baibussinova, Alexandra Lipikhina, Yulia Brait, Alexey Klivenko, Saulesh Apbassova, Gulnara Batenova, Murat Lepesbayev, Saule Kozhanova, Asset Izdenov, Raushan Dosmagambetova, Lyudmila Pivina

**Affiliations:** 1 Office of the Rector, Semey Medical University, Semey 071400, Kazakhstan; altay.dyusupov@smu.edu.kz (A.D.); dosmagambetova@qmu.kz (R.D.); 2 Department of Nursing, Semey Medical University, Semey 071400, Kazakhstan; 3 Department of Public Health, Semey Medical University, Semey 071400, Kazakhstan; 4 Office of the Vice Rector, Semey Medical University, Semey 071400, Kazakhstan; 5 Department of Internal Medicine, Semey Medical University, Semey 071400, Kazakhstan; 6 Research Laboratory Center, Semey Medical University, Semey 071400, Kazakhstan; 7 Department of Biostatistics, Semey Medical University, Semey 071400, Kazakhstan; 8 Research Institute of Radiation Medicine and Ecology, Semey Medical University, Semey 071400, Kazakhstan; 9 Shakarim Lab, Shakarim University, Semey 070000, Kazakhstan; 10 Department of Pathology, Semey Medical University, Semey 071400, Kazakhstan; 11 Department of Emergency Medicine, Semey Medical University, Semey 071400, Kazakhstan; 12 Department of Anatomy, Semey Medical University, Semey 071400, Kazakhstan; 13 Ministry of Health of the Republic of Kazakhstan, Astana 010000, Kazakhstan

**Keywords:** Semipalatinsk Nuclear Test Site, cancer mortality, radiation exposure, Kazakhstan, epidemiology

## Abstract

**Highlights:**

**Public health relevance—How does this work relate to a public health issue?**

- Long-term combined radiation exposure from nuclear weapons testing at the Semipalatinsk nuclear test site led to radioactive contamination of vast areas of Kazakhstan and population exposure.
- Cancer is one of the leading causes of death in radiation-exposed populations and their descendants, necessitating an assessment of its structure and prevalence.

**Public health significance—Why is this work of significance to public health?**

- The relative risk of cancer mortality remains elevated throughout the study period, from the initial period after nuclear weapons testing to the present. Age-standardized mortality rates in people exposed to radiation were significantly higher than those of the unexposed group.
- Among individuals born after the period of aerial and ground nuclear testing, a trend toward increased mortality from cancers of the digestive system and hematological malignancies was found.

**Public health implications—What are the key implications or messages for practitioners, policymakers and/or researchers in public health?**

- The results highlight the importance of further research to examine the association between past radiation exposure and cancer incidence and mortality in the offspring of individuals directly exposed to radiation.
- The findings support the need for screening and in-depth medical studies of individuals exposed to radiation and their offspring to facilitate early detection and prevention of cancer.

**Abstract:**

**Introduction.** Mortality rates from malignant neoplasms can be considered a key marker of adverse environmental impacts. The aim of this study was to assess the association between exposure to ionizing radiation and the risk of mortality from malignant neoplasms among the population of Kazakhstan living in areas adjacent to the Semipalatinsk Nuclear Test Site (SNTS). **Materials and Methods.** The main source of data used in this study was the mortality sub-register of the population living in territories adjacent to the Semipalatinsk Nuclear Test Site (SNTS). The main study group included residents of the Abay District (*n* = 1300) and Beskaragay District (*n* = 414). In total, 478 residents of the Kokpekty District, where the radiation dose was minimal, were included in the control group. The median effective radiation dose was 864 mSv for the population of the exposed group, whereas in the control group, it was 67 mSv. **Results.** The highest RR values were observed in the periods 1965–1969 (RR = 6.14; 95% CI: 4.04–9.34) and 1970–1974 (RR = 6.14; 95% CI: 4.19–8.99). Age-standardized mortality rates (ASMR) in the study group were significantly higher than those in the control group. A dose–response relationship was established: with each 10.0 mSv increase in radiation dose, the risk of cancer mortality increased by 0.9% (OR = 1.009; 95% CI: 1.008–1.010; *p* < 0.001). **Conclusions.** The obtained results indicate a significant association between long-term exposure to ionizing radiation due to testing at the Semipalatinsk Nuclear Test Site and increased cancer mortality among the population of adjacent territories.

## 1. Introduction

The relevance of studying the effects of ionizing radiation on carcinogenesis is driven by the high global incidence and mortality rates of malignant neoplasms. Developed countries tend to have high incidence rates for most cancer types, including lung, colorectal, breast, and prostate cancers, whereas in low-income countries, stomach, liver, esophageal, and cervical cancers predominate. The increase in incidence rates is partly attributable to screening and improved early diagnosis through the introduction of new technologies, including nuclear medicine. At the same time, mortality rates are declining in developed countries due to advances in modern treatment approaches. Meanwhile, in some low- and middle-income countries, cancer incidence continues to rise due to increasing rates of smoking, overweight and obesity, sedentary lifestyles, and the spread of certain infections [1,2,3,4,5].

In addition to established cancer risk factors such as industrial pollution, smoking, exposure to chemicals, and genetic predisposition, ionizing radiation is a well-recognized contributor to carcinogenesis. This is supported by evidence of increased cancer incidence and mortality among workers in the nuclear industry, particularly at facilities involving plutonium exposure. A clear relationship between cancer risk and cumulative doses of α- and β-radiation has been demonstrated [6]. Furthermore, studies of Japanese survivors of the atomic bombings have shown a strong association between radiation exposure and both cancer incidence and mortality [7,8].

The Semipalatinsk Nuclear Test Site (SNTS), located in northeastern Kazakhstan, was the site of 473 nuclear explosions conducted between 1949 and 1989, including 90 atmospheric, 26 surface, and 354 underground tests. The impact of these tests affected vast territories, and the number of affected individuals is estimated in UN documents and official Kazakh sources at approximately 1.5 million people exposed to direct radiation, as well as their descendants [9,10,11]. The relevance of the present study is further supported by the large population residing in regions adjacent to the former SNTS, who continue to live under conditions of environmental stress and experience associated medical and social consequences [12].

To date, only a limited number of studies have assessed the relationship between radiation exposure and the health status of populations in the Semipalatinsk region affected by radioactive fallout. Most of these studies have focused on cardiovascular diseases [13,14,15], while only a few have examined the association between ionizing radiation and cancer outcomes [16,17].

The aim of this study was to assess the association between exposure to ionizing radiation and the risk of mortality from malignant neoplasms among the population of Kazakhstan living in areas adjacent to the Semipalatinsk Nuclear Test Site.

## 2. Materials and Methods

### 2.1. Characteristics of the Study Population

The study was approved by the Ethics Committee of Semey Medical University (Protocol No. 2b, dated 18 December 2024).

This study was a retrospective, longitudinal, ecological time-series study. We calculated age-standardized mortality rates for the radiation-exposed population and controls for five-year periods from 1949 to 2024. The primary data source for this study was the mortality sub-register of the population residing in areas adjacent to the Semipalatinsk Nuclear Test Site (SNTS). This sub-register is part of the State Scientific Automated Medical Registry (SSAMR), established in 2002 with the support of international experts in radiation medicine [18]. The registry includes populations from radiation-exposed areas and was designed to collect their demographic, social, and medical data. One of its key objectives is the long-term monitoring of the health status of radiation-exposed populations and their descendants. Currently, the registry contains data not only on directly exposed individuals but also on second- and third-generation descendants, enabling a more comprehensive assessment of the long-term health effects of radiation exposure in Kazakhstan.

In total, the registry includes more than 370,000 citizens of the Republic of Kazakhstan, each assigned a unique identification number that allows access to all relevant data within the system [18]. As of 2025, the mortality sub-register contains data on 11,955 individuals, of whom 2192 (18.3%) deaths were attributable to cancer.

The mortality sub-register covers the period from 1949 to 2024. It included all people who lived in the studied areas at the time of death. The database was compiled through case-by-case extraction and verification of information from regional civil registration offices (ZAGS), the Unified Medical Information System of the Republic of Kazakhstan, and administrative records from local akimats (government agencies). Population data for the study areas were obtained from the Bureau of National Statistics of the Republic of Kazakhstan and archival sources [19].

The main source of information was death certificates completed by the attending physician and having the status of an official document. For each case, the following information was available: date of birth and death, age, sex, ethnicity, occupation, cause of death (coded according to the International Classification of Diseases, 10th Revision (ICD-10)), place of permanent residence, and radiation dose (mSv). Causes of death were coded by two qualified medical specialists.

The main study group included residents of the Abay District (villages of Karaul, Sarzhal, and Kainar; *n* = 1300) and Beskaragay District (villages of Dolon, Mostik, and Cheremushka; *n* = 414). The primary indicator for selecting participants in this group was the radiation exposure dose, which was approximately equivalent for residents of both districts. This allowed us to combine residents of the two districts into a radiation-exposed group. 478 residents of the Kokpekty District, where the radiation dose was minimal, were included in the control group.

The study was approved by the Ethics Committee of Semey Medical University (protocol No. 2b, 18 December 2024).

### 2.2. Estimation of Radiation Exposure Doses

**Figure 1:**
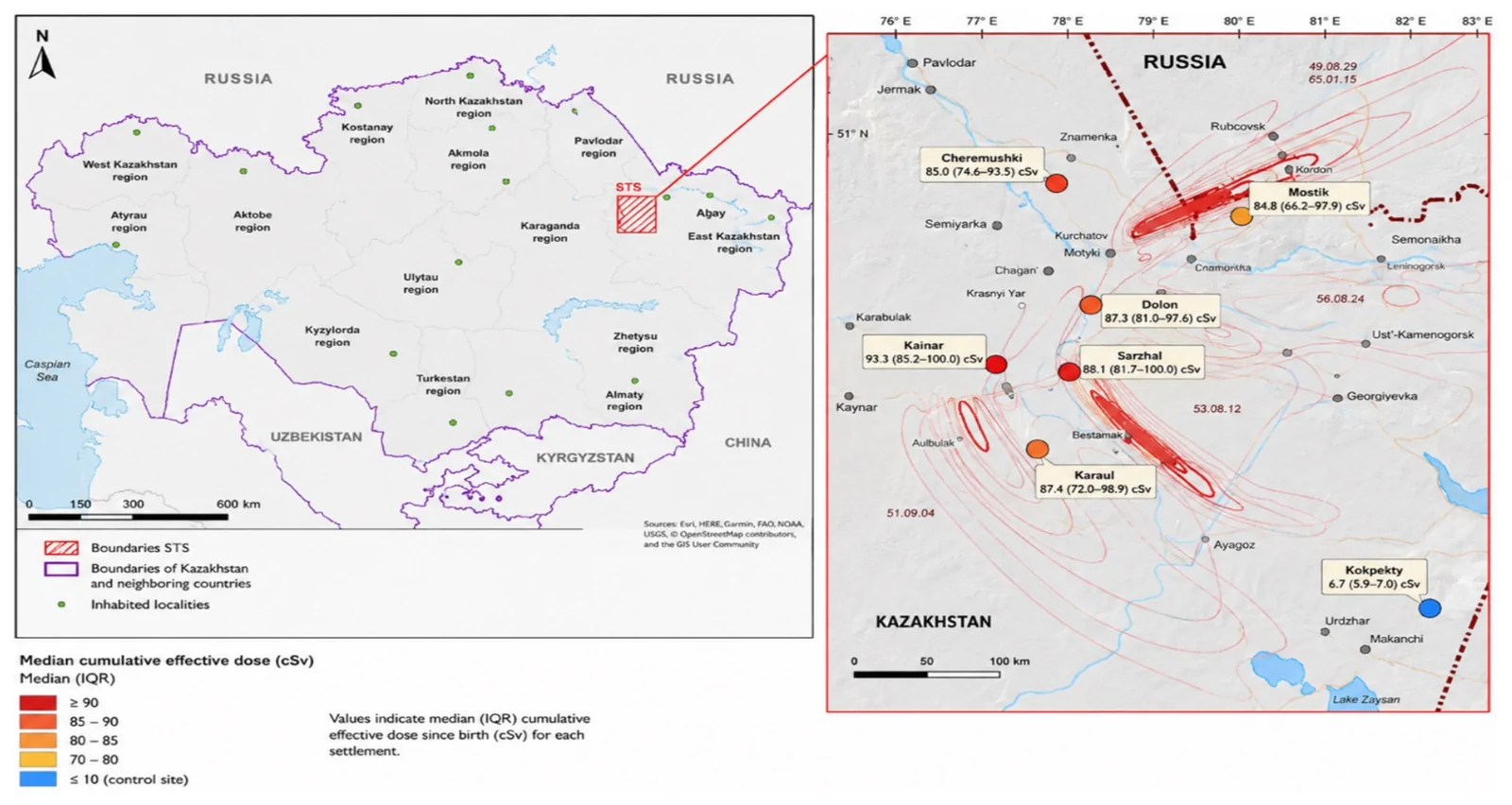
Distribution of accumulated effective radiation doses to the population of the studied settlements located near the Semipalatinsk test site.

The median cumulative effective radiation doses (mSv) are shown in color; the values in parentheses correspond to the interquartile range (Q1–Q3). The red contours indicate the zones of radioactive contamination resulting from atmospheric nuclear tests (Figure 1).

The Semipalatinsk test site is located on the border of three regions of Kazakhstan: Semipalatinsk in the east, Pavlodar in the north, and Karaganda in the west. The red lines indicate the directions of radioactive traces from the main dose-forming explosions.

Individual radiation doses were calculated in accordance with official methodological guidelines adopted in the Republic of Kazakhstan, using specialized software integrated into the SSAMR system [20].

The median effective radiation dose was 888.0 mSv (IQR: 771.0–1000.0 mSv) for the population of the Abay District and 860.0 mSv (IQR: 778.0–971.0 mSv) for the Beskaragay District, whereas in the Kokpekty District (control group), it was 67.0 mSv (IQR: 59.0–70.0 mSv).

According to the Methodological Recommendations of the Republic of Kazakhstan [20], the fundamental criterion for individual dose assessment is the determination of the contribution of both external gamma radiation exposure and internal radiation exposure resulting from the intake of long-lived radionuclides into the human body through inhalation of contaminated air during the passage of radioactive clouds, as well as through the consumption of contaminated drinking water and locally produced food products. Dose assessment used dosimetry methodology with settlement-specific exposure rate values (the yield and type of explosions (surface and underground), detonation height, wind speed, climatic conditions, radioactive plume trajectories, and fallout arrival times) that were measured or reconstructed. Calculation of individual radiation doses takes into account each participant’s date of birth, age-specific behavioral factors, occupation, and residential history, including the end date of residence in each settlement, corresponding either to migration from the settlement or to death [21,22,23,24].

Of the atmospheric nuclear weapons tests conducted at the SNTS between 1949 and 1962, Gordeev et al. (2002) identified 11 tests that led to radiation exposure among residents of settlements in northeastern Kazakhstan [23]. Dose reconstruction included all settlements in which study participants resided during the period 1949–1990 and which were affected by radioactive fallout resulting from nuclear weapons testing at the SNTS. Individual cumulative doses were calculated for the period from the beginning of radiation exposure (i.e., the arrival of radioactive fallout in the settlement of residence, the individual’s date of birth, or the date of arrival in a radiation-affected settlement) until the end of radiation exposure (i.e., cessation of nuclear testing, departure from the radiation-affected settlement, or death).

When assessing radiation doses, only external radiation doses from the explosions of 1949–1962 were taken into account, as the contribution of internal radiation to individual radiation doses cannot be retrospectively assessed at this time, and no similar dosimetric studies are available in the scientific literature.

Residents of the Kokpekty district, included in the control group, received a radiation dose during the same period of open nuclear testing, from 1949 to 1962.

### 2.3. Statistical Analysis

Statistical analyses were conducted using SPSS (version 20), R software (version 4.4.1), and Jamovi (version 2.5). Continuous variables were tested for normality using the Shapiro–Wilk test and are presented as median and interquartile range (IQR) because most variables were not normally distributed. Comparisons between groups were performed using the Mann–Whitney U test for continuous variables and the χ^2^ test for categorical variables. Cancer mortality rates were calculated separately for the exposed and comparison populations. Age-standardized mortality rates (ASMRs) per 100,000 population were estimated using the World Health Organization (WHO) World Standard Population. Relative risks (RRs) and corresponding 95% confidence intervals (95% CIs) were calculated to compare cancer mortality between radiation-exposed and comparison populations across different calendar periods.

To assess the association between cumulative radiation dose and cancer mortality, binary logistic regression models with a dependent variable (death from cancer) were constructed. Results are presented as odds ratios (ORs) with 95% confidence intervals for each 1 mSv increase in dose.

All statistical tests were two-sided, and *p*-values < 0.05 were considered statistically significant.

## 3. Results

The sociodemographic characteristics of the study participants who died from malignant neoplasms are presented in Table 1. The groups were comparable by gender (*p* = 0.133) but differed in ethnic composition and age. The study group had a higher proportion of people of Asian origin (82.3% vs. 64.2%; *p* < 0.001), and the median age at death was lower than in the control group (61 (53–69) vs. 65 (55–73) years; *p* = 0.001).

Table 2 presents the effective cumulative radiation dose in the exposed and control groups. When analyzing the accumulated radiation dose, a statistically significant difference was found in the study groups depending on the date of birth. Thus, the maximum radiation dose was established among individuals born before 1962 (890.0 (810.0–1000.0) and 67.0 (60.7–70.0) mSv in the main and control groups, respectively), that is, during the period of above-ground nuclear tests on the territory of the SNTS. Subsequently, the radiation dose decreased and amounted to 132.0 (62.9–159.0) and 11.3 (8.08–13.4) mSv, respectively. Among individuals born after the cessation of the tests, the radiation dose was not verified by the calculation method.

**Figure 2:**
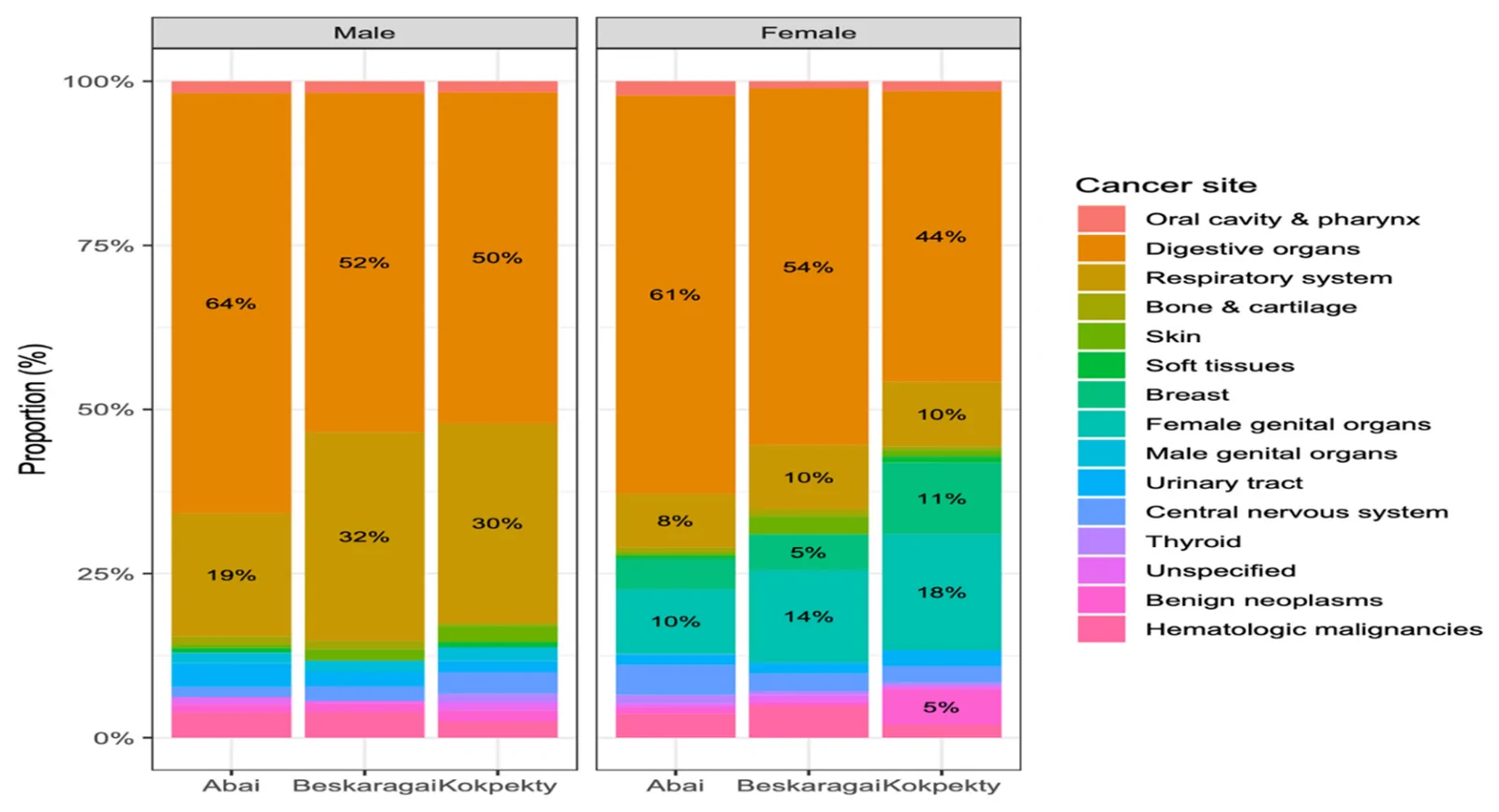
Structure of cancer mortality in the studied areas depending on gender.

The structure of cancer mortality varied by gender and region of residence (Figure 2). In all regions, malignant neoplasms of the digestive system accounted for the largest proportion of deaths. Among men, their share reached 64.0% in the Abay District, approximately 53–55% in the Beskaragay District, and less than 50% in the Kokpekty District. A similar pattern was observed among women, although the proportion was somewhat lower. Among men, malignant neoplasms of the respiratory system ranked second. In the Abay District, their proportion was 18.8%, while in the Beskaragay and Kokpekty districts, it was slightly higher, reaching approximately 20–22%. Among women, the proportion of these tumors was considerably lower, typically not exceeding 10–15%. In female mortality, a higher proportion of malignant neoplasms of the breast and female genital organs was observed, together accounting for a substantial share of deaths (up to 10–15%). Among men in the Abay District, malignant neoplasms of the urinary tract accounted for 3.6% of deaths, while tumors of the central nervous system accounted for 1.5%. Similar values were observed in the other districts, with no statistically significant differences. Hematological malignancies accounted for approximately 3–5% of deaths and tended to be more frequent in the Beskaragay District. Rare localizations (bone, soft tissue, thyroid, and other tumors) collectively accounted for less than 3% of deaths and showed no significant differences between districts.

In all studied time periods, the relative risks (RRs) of mortality from cancer for people exposed to radiation were high. The highest RR values were observed in the periods 1965–1969 (RR = 6.14; 95% CI: 4.04–9.34) and 1970–1974 (RR = 6.14; 95% CI: 4.19–8.99). Age-standardized mortality rates (ASMRs) in the study group were significantly higher than those in the control group (Table 3).

**Figure 3:**
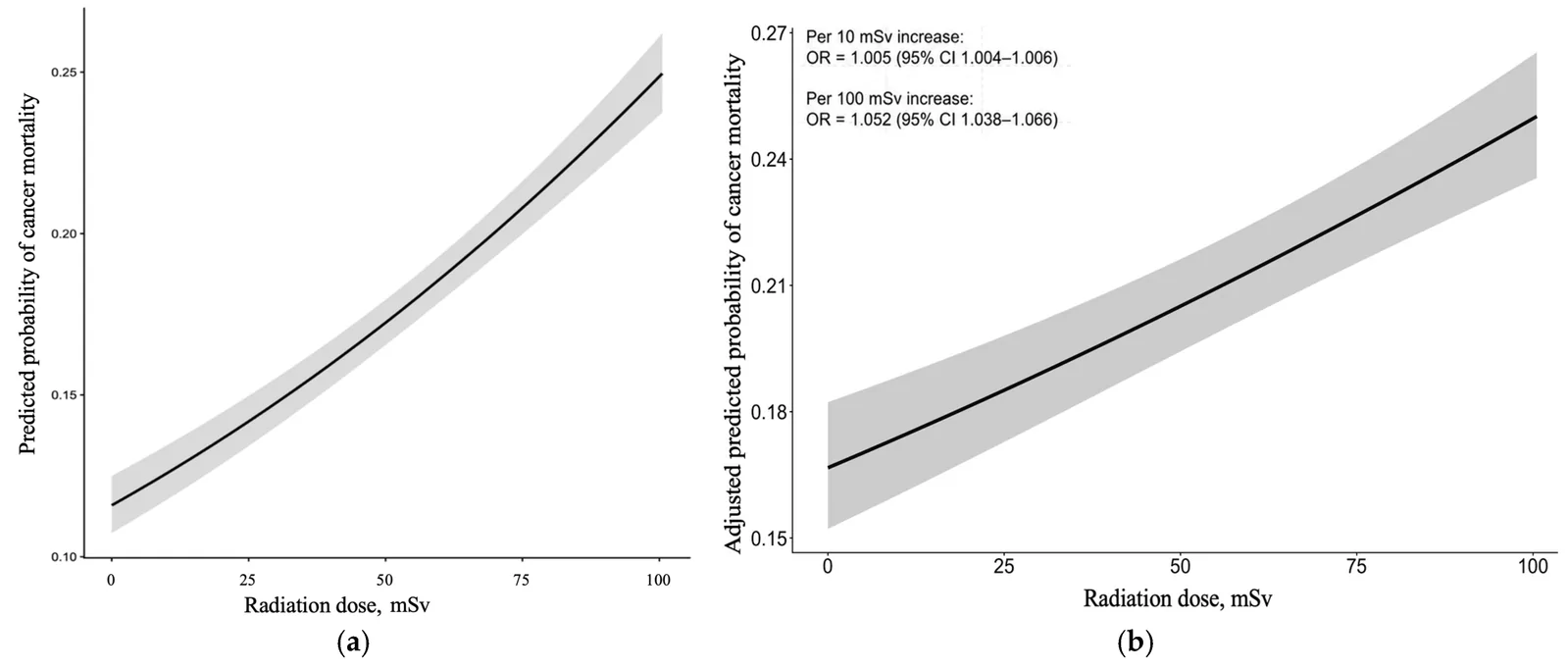
(**a**) Dose–response relationship between cumulative radiation dose and the predicted probability of cancer-related death in the population of the study areas, based on logistic regression data. (**b**) Adjusted relationship between cumulative radiation dose (mSv) and the probability of death from cancer, obtained using multivariate logistic regression. The dark area indicates the 95% CI. ORs are given for each 10 mSv increase in radiation dose.

Logistic regression analysis revealed a statistically significant association between cumulative radiation dose and the odds of cancer-related death (Figure 3). For every 10 mSv increase in radiation dose, the odds of cancer-related death increased by 0.9% (OR = 1.009; 95% CI 1.008–1.010; *p* < 0.001) (Figure 3a). When expressed per 100 mSv increase, the corresponding odds ratio was 1.094 (95% CI 1.083–1.105), indicating that the cumulative effect becomes more apparent over dose increments representative of the study population.

We also adjusted the resulting dose–response model for age at death, gender, and birth cohort (Figure 3b). After adjustment for age at death, sex, and birth cohort, a statistically significant positive association remained between cumulative radiation dose and the odds of cancer-related death. The predicted odds of cancer mortality increased from 16.7% at a dose close to 0 mSv to 25.0% at a dose of 1000.0 mSv. According to the multivariable logistic regression model, cumulative radiation dose remained an independent predictor of cancer-related death. For every 10 mSv increase in cumulative radiation dose, the odds of cancer-related death increased by 0.5% (OR = 1.005; 95% CI 1.004–1.006; *p* < 0.001). When recalculated for every 100 mSv, the odds ratio was 1.052 (95% CI 1.038–1.066). Given the median radiation dose for individuals in the main study group of 864 mSv, one would expect an approximate increase in the chance of cancer mortality in this group of 22–23%.

The results of binary logistic regression (Table 4) showed that the unadjusted OR of death revealed statistically significant associations of radiation dose with mortality from cancer of the digestive system (OR 1.008; 95% CI 1.006–1.010), as well as hematological malignancies (OR 1.011; 95% CI 1.008–1.014). However, for some cancers, a statistically significant association remained after adjustment for age. For example, for gastrointestinal cancer, increasing radiation dose was associated with an increased likelihood of death (OR = 1.005; 95% CI: 1.002–1.007). In contrast, for gynecological cancer (OR = 0.993; 95% CI: 0.988–0.998) and breast cancer (OR = 0.983; 95% CI: 0.976–0.990), an inverse statistically significant association between dose and risk of death was observed. These results may reflect differences in the demographic structure of the study groups, competing causes of death, or limitations in retrospective mortality registration, rather than indicating a protective effect of radiation exposure.

Overall, these findings indicate that a positive dose–response association was primarily observed for digestive system cancers and hemoblastoses, whereas associations for other cancer sites were either absent or inconsistent after age adjustment.

Given that ground-based and airborne nuclear testing was banned only in 1962, it was of significant interest to us to analyze the structure of malignant neoplasm localizations depending on the birth period of individuals included in the main study group. The analysis revealed an unequal structure of causes of cancer mortality between groups depending on the birth period (Table 5). Digestive system cancers remained the most common cause of death in all groups; however, their proportion was significantly higher among individuals born before 1962 (62.3%) compared to individuals born in 1963–1989 (24.3%) and after 1990 (0.0%; *p* < 0.001). The proportion of deaths from brain cancer also differed between the groups (2.3%, 9.5%, and 28.6%, respectively; *p* < 0.001), as did the proportion of hematological malignancies (3.4%, 16.2%, and 0.0%; *p* < 0.001). In addition, statistically significant differences were found for breast cancer (1.7%, 8.1%, and 0.0%; *p* < 0.001) and urinary tract cancer (2.3%, 6.8%, and 14.3%; *p* = 0.009). For the remaining localizations, no statistically significant differences were found between the groups (all *p* > 0.05). It should be noted that the group of people born after 1990 was small, so the results for this group should be interpreted with caution.

Binary logistic regression analysis stratified by year of birth revealed differences in the nature of the associations between cumulative radiation dose and mortality from malignant neoplasms of different localizations (Table 6). Among people born before 1962, after adjustment for multiple comparisons, associations with cumulative radiation dose persisted for urinary tract cancer (OR = 1.040; 95% CI: 1.010–1.070; *p*-adjusted FDR = 0.033), respiratory cancer (OR = 1.008; 95% CI: 1.001–1.016; *p*-adjusted FDR = 0.035), breast cancer (OR = 0.978; 95% CI: 0.965–0.990; *p*-adjusted FDR = 0.010), and hematological malignancies (OR = 0.986; 95% CI: 0.976–0.996; *p*-adjusted FDR = 0.030). Among individuals born after 1963, after adjusting for multiple comparisons, no statistically significant associations were found between cumulative radiation dose and mortality for any of the malignant neoplasm sites studied. However, trends toward association were observed for breast cancer (*p* = 0.069), gastrointestinal cancer (*p* = 0.059), and hematological malignancies (*p* = 0.082); however, after adjusting for the false discovery rate (FDR), these did not reach statistical significance.

## 4. Discussion

Cancer mortality is widely used as a key indicator of the impact of ionizing radiation on public health. The relationship between radiation exposure and cancer has been extensively studied in the context of major radiation events, including the atomic bombings of Hiroshima and Nagasaki, the Chernobyl nuclear power plant accident, and occupational exposure during nuclear weapons production [25,26,27,28,29,30]. Cancer mortality reflects the long-term health effects of ionizing radiation in exposed populations. Moreover, cause-of-death data are derived from death certificates issued by certified professionals, making them reliable and accessible for epidemiological analysis. Mortality rates can also be analyzed over time, allowing the identification of latency periods for different cancer sites.

However, several limitations should be considered. The long latency period between exposure and cancer development (often up to 30 years) complicates causal inference. In addition, multiple confounding factors—including smoking, occupational exposures, diet, water quality, and other environmental influences—may affect cancer mortality patterns [31]. It is also important to consider that radiosensitivity varies across cancer types. For example, thyroid cancer and acute leukemia are highly radiosensitive and typically have shorter latency periods, whereas many solid tumors are less radiosensitive and develop over longer periods [32].

According to Brenner et al., analysis of the Life Span Study (LSS) cohort of Hiroshima and Nagasaki atomic bomb survivors (1958–2009) demonstrated differences between cancer incidence and mortality structures. More lethal cancers (e.g., liver, pancreas, lung, and other gastrointestinal cancers) were more prevalent in mortality data, whereas less lethal cancers (e.g., colorectal cancer, brain tumors, and thyroid cancer) were more prominent in incidence data. Dose–response relationships were generally consistent for both incidence and mortality, although nonlinearity varied by sex and time period [33,34,35,36].

Among workers at the Fernald Raw Materials Production Center uranium processing plant, a statistically significant dose–response relationship was found between external radiation dose and lung cancer mortality (at 100 mGy, RR = 1.45; 95% CI = 1.05–2.01) [37].

In our study, the population living near the Semipalatinsk test site received substantially higher cumulative radiation doses (median 864.0 mSv in the exposed group) due to radiation exposure. These levels exceed those reported for many previously studied populations. An analysis of demographic data reveals a statistically significant younger age of onset of cancer death among individuals exposed to radiation; Kazakhs predominated among them. Those born during the period of above-ground nuclear testing received the highest radiation dose.

The structure of cancer mortality in our study is consistent with global evidence. Gastrointestinal cancers predominated in all districts. Respiratory cancers ranked second among men, while breast and female genital cancers were the second most common among women. Hematological malignancies accounted for approximately 3–5% of deaths but demonstrated notably elevated relative risks in exposed populations.

The relative risks of mortality from cancer for people exposed to radiation were high for the entire period of study (RR from 6.14 in the initial period to 2.21 in the last decade). Age-standardized mortality rates were also significantly higher than those of the unexposed group.

We identified a dose–response relationship, with a 0.9% increase in cancer mortality risk for every 10.0 mSv increase in radiation dose. No significant gender differences in cancer mortality risk were observed. After adjusting for age at death, a statistically significant positive association between cumulative radiation dose and the probability of death from digestive system cancers and hematological malignancies remained. Regression analysis of the relationship between radiation dose and individual cancer sites showed a statistically significant increase in the odds of mortality from urinary tract cancer, respiratory system cancer, and hematological malignancies in the group of people born before 1962. However, in the group of people born after the period of airborne and ground-based nuclear testing, we found only a trend toward an increase in the odds of mortality from cancers of the digestive system and hematological malignancies. For breast cancer and female reproductive system cancers, we did not find a statistically significant positive association with radiation dose. This can be explained by the low number of deaths from these cancer sites (34 and 77 cases, respectively) and the relatively favorable prognosis of breast cancer compared to other cancer sites due to better diagnostics. The small number of cancer cases in these sites can be considered a limitation of our study; therefore, the observed negative association with radiation dose should not be interpreted as a protective effect of radiation exposure. Furthermore, a cohort study could be a more accurate tool for tracking causes of death and their number, with a more precise determination of the duration of residence in radiation-contaminated areas.

In a recently published systematic review with meta-analysis of breast cancer incidence and mortality in population studies of radiation exposure [38], the authors found that radiation exposure was associated with increased breast cancer risk (ERR/Gy = 0.56, 95% CI: 0.29–0.83). However, this systematic review included studies on both mortality and incidence of breast cancer, and the studies were significantly heterogeneous; some environmental studies have shown a negative association between breast cancer and radiation dose [38].

The only previous large-scale cohort study of cancer mortality in populations exposed to fallout from the Semipalatinsk nuclear test site (Bauer et al., 2005) also demonstrated significant dose–response relationships for all solid cancers, as well as for gastrointestinal and respiratory system cancers [16]. A dose–response relationship was found in the exposed group for all solid cancers (*p* < 0.0001), as well as for cancer of the digestive system, lung cancer (*p* = 0.0255 and *p* < 0.0001, respectively), stomach cancer (*p* = 0.0050), breast cancer in women (*p* = 0.0040), and esophageal cancer in women (*p* = 0.0030). The excess relative risk per sievert for all solid tumors combined was 1.77. Esophageal cancer was found substantially elevated, with a statistically significant increase in the relative risk with dose and an ERR/Sv of 2.37 (1.45; 3.28) for the total cohort. These findings are consistent with our results.

Comparisons with other populations are limited due to the scarcity of relevant studies. For example, estimated radiation doses from nuclear weapons testing in Nevada were substantially lower (approximately 0.5 mGy external exposure), and data on long-term cancer mortality in those populations remain limited [39,40]. Evidence from the LSS cohort further supports a linear dose–response relationship for solid cancers across a wide dose range, with higher risks observed among individuals exposed at younger ages [41,42,43]. In contrast to LSS findings, our study did not demonstrate a significant increase in thyroid cancer mortality, which may be explained by differences in exposure patterns. Unlike the acute exposure in Japan, the population near the Semipalatinsk test site experienced prolonged combined external and internal exposure. Additionally, thyroid cancer is characterized by relatively low mortality despite high incidence, as demonstrated in post-Chernobyl studies [44]. Our findings are also consistent with data from Chernobyl cleanup workers, where gastrointestinal, lung, and hematological malignancies constituted a substantial proportion of cancer mortality [45].

### Limitations of the Study

Several limitations of this study should be recognized. The retrospective design precluded estimation of excess relative risk per unit dose. Variability in diagnostic practices, disease classification systems (transition from ICD-8 to ICD-10), and healthcare access over the extended study period (1949–2024) may also have influenced mortality patterns. The study had a retrospective, longitudinal, ecological time-series design, although a cohort study would have been the most appropriate design. Nevertheless, this study represents one of the first comprehensive population-level assessments of cancer mortality in regions affected by prolonged radioactive fallout. Death certificates contained information on the patient’s age, education, marital status, and diagnosis. Insufficient information prevented us from assessing the contribution of all traditional risk factors to cancer mortality in the study population, except for age. This fact determined the choice of binary logistic regression to assess the association of radiation dose with the effect under study.

## 5. Conclusions

This study demonstrates an association between long-term exposure to ionizing radiation from nuclear weapons testing at the Semipalatinsk test site and increased cancer mortality. A dose–response relationship was identified, with a 0.9% increase in mortality risk per 10 mSv. Elevated mortality rates were observed primarily during earlier decades (1949–1979) in exposed populations. The highest ORs were associated with cancers of the digestive system and hematological malignancies. These findings highlight the need for targeted public health strategies in Kazakhstan, including long-term monitoring, prevention programs, and rehabilitation measures for affected populations.

## Figures and Tables

**Table 1:** Sociodemographic characteristics of the study groups.

Rate	Total, N (%)	Exposed Group, N (%)	Control Group, N (%)	*p*
Gender				
Male	1248 (57.9)	1001 (58.7)	247 (54.6)	0.133
Female	909 (42.1)	704 (41.3)	205 (45.4)	
Nationality				
Asians	1693 (78.5%)	1403 (82.3%)	290 (64.2%)	<0.001
Europeans	464 (21.5%)	302 (17.7%)	162 (35.8%)	
Age (years), Mean (Q1–Q3)	64.0 (54.0 to 72.0)	61.0 (53.0 to 69.0)	65.0 (55.0 to 73.0)	0.001

**Table 2:** Cumulative effective radiation doses to the studied population (mSv).

Groups	Exposed Group	Control Group	*p*
Total	864.0 (376.0–980.0)	67.0 (59.0–70.0)	<0.001
Born before 1962	890.0 (810.0–1000)	67.0 (60.7–70.0)	<0.001
1963–1989	132.0 (62.9–159.0)	11.3 (8.08–13.4)	<0.001
After 1990	0	0	-

**Table 3:** Age-standardized mortality rate and relative risks (per 100,000 population).

Period	ASMR in the Exposed Group	ASMR in the Control Group	RR	95% CI
1949–54	186.5	36.9	5.05	2.49–10.25
1955–59	155.4	36.9	4.21	2.19–8.10
1960–64	345.0	88.0	3.92	2.60–5.91
1965–69	440.5	71.7	6.14	4.04–9.34
1970–74	422.6	68.8	6.14	4.19–8.99
1975–79	304.9	80.0	3.81	2.73–5.32
1980–84	277.3	131.1	2.12	1.63–2.75
1985–89	263.5	113.3	2.33	1.81–3.00
1990–94	263.5	160.2	1.65	1.30–2.10
1995–99	251.9	94.0	2.68	2.01–3.58
2000–04	340.0	164.5	2.07	1.64–2.61
2005–09	349.9	272.6	1.28	1.02–1.61
2010–14	245.5	156.0	1.57	1.16–2.11
2015–19	290.2	143.2	2.03	1.30–3.16
≥2020	337.5	152.5	2.21	1.39–3.51

**Table 4:** Binary logistic regression to assess the relationship between radiation dose and mortality from cancer of various localizations.

Localization of Cancer	Unadjusted OR (95% CI)	*p*	*p* *	Age-Adjusted OR (95% CI)	*p*	*p* *
Thyroid cancer	0.989 (0.975–1.003)	0.234	0.390	0.991 (0.976–1.006)	0.792	0.792
Brain cancer	0.990 (0.984–0.997)	<0.001	0.0025	0.998 (0.990–1.005)	0.154	0.240
Urinary tract cancer	1.003 (0.995–1.010)	0.765	0.850	1.003 (0.995–1.011)	0.724	0.792
Cancer of the male reproductive system	0.992 (0.981–1.0028)	0.44	0.629	0.990 (0.979–1.000)	0.06	0.150
Cancer of the female reproductive system	0.999 (0.99–1.007)	0.573	0.716	0.993 (0.988–0.998)	0.02	0.792
Breast cancer	0.979 (0.969–0.989)	<0.001	0.0025	0.983 (0.976–0.990)	<0.001	0.0033
Skin cancer	0.993 (0.983–1.004)	0.994	0.994	0.990 (0.980–1.001)	0.168	0.240
Digestive system cancer	1.008 (1.006–1.010)	<0.001	0.0025	1.026 (1.018–1.034)	<0.001	0.0033
Respiratory cancer	1.000 (0.997–1.003)	0.015	0.030	1.000 (0.997–1.003)	0.121	0.240
Hematological malignancies	1.011 (1.008–1.014)	<0.001	0.0025	1.007 (1.003–1.011)	<0.001	0.0033

OR, odds ratio; CI, confidence interval; FDR, false discovery rate. * FDR-adjusted *p*-values were calculated using the Benjamini–Hochberg procedure.

**Table 5:** Distribution of localizations of malignant neoplasms depending on the group by year of birth.

Localization of Cancer	Born Before 1962, N (%)	Born Between 1963 and 1989, N (%)	Born After 1990, N (%)	*p*
Thyroid cancer	8 (0.5)	0 (0.0)	0 (0.0)	χ^2^ = 0.40, *p* = 0.821
Brain cancer	37 (2.3)	7 (9.5)	2 (28.6)	χ^2^ = 31.809, *p* < 0.011 *p* * 1–2 < 0.012 *p* * 1–3 < 0.012 *p* * 2–3 = 0.0092
Urinary tract cancer	38 (2.3)	5 (6.8)	1 (14.3)	χ^2^ = 9.32, *p* = 0.0091 *p* * 1–2 = 0.0572 *p* * 1–3 = 0.142 *p* * 2–3 = 0.692
Cancer of the male reproductive system	14 (0.9)	2 (2.7)	0 (0.0)	χ^2^ = 2.64, *p* = 0.271
Cancer of the female reproductive system	72 (4.4)	5 (6.8)	0 (0.0)	χ^2^ = 1.22, *p* = 0.541
Breast cancer	28 (1.7)	6 (8.1)	0 (0.0)	χ^2^ = 14.90, *p* < 0.011 *p* * 1–2 < 0.012 *p* * 1–3 = 1.0 *p* * 2–3 = 0.428
Skin cancer	15 (0.9)	0 (0.0)	0 (0.0)	χ^2^ = 0.75, *p* = 0.691
Digestive system cancer	1011 (62.3)	18 (24.3)	0 (0.0)	χ^2^ = 53.22, *p* < 0.011 *p* * 1–2 < 0.012 *p* * 1–3 = 0.0022 *p* * 2–3 = 0.6262
Respiratory cancer	267 (16.4)	10 (13.5)	1 (14.3)	χ^2^ = 0.46, *p* = 0.791
Bone cancer	17 (1.0)	2 (2.7)	0 (0.0)	χ^2^ = 1.84, *p* = 0.391
Hematological malignancies	55 (3.4)	12 (16.2)	0 (0.0)	χ^2^ = 31.127, *p* < 0.011 *p* * 1–2 < 0.012 *p* * 1–3 = 1.02 *p* * 2–3 = 0.1052

*p*—Pearson’s chi-square (χ^2^) test; when the expected cell frequency was <5, Fisher’s exact test was used. *p* *—Bonferroni-adjusted *p*-value.

**Table 6:** Binary logistic regression analysis of the association between radiation dose and site-specific cancer mortality by birth groups.

Localization of Cancer	OR (95% CI), Born Before 1962	*p*	*p* *	OR (95% CI), Born After 1962	*p*	*p* *
Thyroid cancer	0.978 (0.955–1.002)	0.067	0.112	-	-	–
Brain cancer	0.986 (0.974–0.999)	0.028	0.056	0.926 (0.83–1.032)	0.163	0.223
Urinary tract cancer	1.04 (1.01–1.07)	0.01	0.033	0.896 (0.78–1.027)	0.113	0.223
Cancer of the male reproductive system	0.997 (0.973–1.023)	0.857	0.916	1.009 (0.82–1.242)	0.929	0.929
Cancer of the female reproductive system	0.999 (0.9887–1.01)	0.916	0.916	1.174 (0.935–1.476)	0.167	0.223
Breast cancer	0.978 (0.965–0.99)	<0.001	0.010	1.283 (0.981–1.679)	0.069	0.219
Skin cancer	0.993 (0.972–1.014)	0.543	0.679	-	-	–
Digestive system cancer	1.0 (0.997–1.01)	0.435	0.621	1.095 (0.99–1.204)	0.059	0.219
Respiratory cancer	1.008 (1.001–1.016)	0.014	0.035	1.043 (0.93–1.16)	0.426	0.487
Hematological malignancies	0.986 (0.976–0.996)	0.006	0.030	0.917 (0.832–1.01)	0.082	0.219

FDR-adjusted *p*-values were calculated separately for the birth cohort-specific analyses using the Benjamini–Hochberg procedure. *p* *—Bonferroni-adjusted *p*-value.

## Data Availability

The raw data supporting the conclusions of this article will be made available by the authors upon request.
